# Comment on “The effect of disease-modifying anti-rheumatic drugs on skeletal muscle mass in rheumatoid arthritis patients: a systematic review with meta-analysis”

**DOI:** 10.1186/s13075-022-02931-6

**Published:** 2022-11-01

**Authors:** Hiroto Minamino, Mie Torii, Masao Katsushima, Yoshihito Fujita, Motomu Hashimoto

**Affiliations:** 1 Department of Diabetes, Endocrinology and Nutrition, Graduate School of Medicine, Kyoto University, 54 Shogoin, Kawahara-cho, Sakyo-ku, Kyoto-shi, Kyoto, 606-8507 Japan; 2 Department of Human Health Sciences, Graduate School of Medicine, Kyoto University, 54 Shogoin, Kawahara-cho, Sakyo-ku, Kyoto-shi, Kyoto, 606-8507 Japan; 3 Department of Rheumatology and Clinical Immunology, Graduate School of Medicine, Kyoto University, 54 Shogoin, Kawahara-cho, Sakyo-ku, Kyoto-shi, Kyoto, 606-8507 Japan; 4 Department of Clinical Immunology, Graduate School of Medicine, Osaka Metropolitan University, 1-4-3, Asahi-machi, Abeno-ku, Osaka, 545-8586 Japan

**Keywords:** Rheumatoid arthritis, Skeletal muscle mass, Sarcopenia, DMARD, Meta-analysis

## Abstract

We read with great interest the article by Hein et al., which described the meta-analysis study on the impact of disease-modifying anti-rheumatic drugs (DMARDs) therapy on skeletal muscle mass in rheumatoid arthritis (RA) patients. While the data presented are impressive, we add some remarks about methodological issues that should be considered. First, this meta-analysis does not include several necessary studies that have provided data on the relationship between anti-tumor necrosis factor (anti-TNF) therapy and body composition. To make the meta-analysis more comprehensive, it could be necessary to incorporate these studies into this analysis. Second, this study did not employ a representative measure of skeletal muscle mass that was adjusted for body size, such as skeletal muscle mass index (SMI). It is well recognized that skeletal muscle mass varies with body size, particularly height and body mass index. Given the heterogeneity background of body size in the studies included in this meta-analysis, it may be worthwhile to conduct an additional analysis regarding the associations between DMARDs and the adjusted measure of skeletal muscle mass such as SMI, which is recommended in several guidelines when determining and contrasting the quantity of skeletal muscle mass. Third, when determining body composition, several reports show variances between bioelectrical impedance analysis (BIA) and dual-energy X-ray absorptiometry (DEXA) in RA as well as in general. In this regard, it may not be appropriate to simultaneously perform a meta-analysis of skeletal muscle mass determined by DEXA and BIA. With the issues described above, we conclude by recommending additional investigations to strengthen the arguments presented by this valuable meta-analysis.

Dear Editor,

We read with great interest the article by Hein et al. [1], which described the meta-analysis study on the impact of disease-modifying anti-rheumatic drugs (DMARDs) therapy on skeletal muscle mass in rheumatoid arthritis (RA) patients. While the data presented are impressive, we would like to add some remarks about methodological issues that should be considered.

First, this meta-analysis does not include several necessary studies that have provided data on the relationship between anti-tumor necrosis factor (anti-TNF) therapy and body composition. For instance, Serelis et al. showed the information on body composition before and one year following anti-TNF therapy [2]. Additionally, Eric Toussirot et al. also presented data on body composition before, 1 year after, and 2 years after anti-TNF medication [3]. These studies appear to meet the criteria proposed by the authors; thus, it could be necessary to include them to make the meta-analysis more comprehensive.

Second, this study did not employ a representative measure of skeletal muscle mass that was adjusted for body size, such as skeletal muscle mass index (SMI). It is well recognized that skeletal muscle mass varies with body size, particularly height and body mass index (BMI). This raises the possibility that even when the same proportion of skeletal muscle mass changes, the absolute degree of that change may vary between populations with larger and smaller body sizes. For this reason, several recommendations, including those for sarcopenia, recommend using skeletal muscle mass that has been adjusted for height or BMI when determining and contrasting the quantity of skeletal muscle mass [4, 5]. Given the heterogeneity background of the studies included in this meta-analysis, it may be worthwhile to conduct an additional analysis regarding the associations between DMARDs and the adjusted indicator of skeletal muscle mass such as SMI, which indicators are included in several studies in this meta-analysis.

Third, when determining body composition, there are variances between bioelectrical impedance analysis (BIA) and dual-energy X-ray absorptiometry (DEXA). In comparison to DEXA, BIA is known to overestimate skeletal muscle mass in the general population [6]. Similar to this, BIA has overestimated the amount of fat-free mass in RA patients [7]. In this regard, it may not be appropriate to simultaneously perform a meta-analysis of skeletal muscle mass determined by DEXA and BIA.

We conclude by recommending more investigations to strengthen the arguments presented by this informative meta-analysis due to the significant methodological issues we raised.

## Data Availability

Not applicable.

## Abbreviations

DMARDs: Disease-modifying anti-rheumatic drugs
RA: Rheumatoid arthritis
TNF: Tumor necrosis factor
SMI: Skeletal muscle mass index
BMI: Body mass index
DEXA: Dual-energy X-ray absorptiometry
BIA: Bioelectrical impedance analysis
